# Author Correction: Supply chains create global benefits from improved vaccine accessibility

**DOI:** 10.1038/s41467-023-41336-0

**Published:** 2023-09-06

**Authors:** Daoping Wang, Ottar N. Bjørnstad, Tianyang Lei, Yida Sun, Jingwen Huo, Qi Hao, Zhao Zeng, Shupeng Zhu, Stéphane Hallegatte, Ruiyun Li, Dabo Guan, Nils C. Stenseth

**Affiliations:** 1 Department of Computer Science and Technology, University of Cambridge, Cambridge, UK; 2 The World Economic Forum, Geneva, Switzerland; 3 Center for Infectious Disease Dynamics, Department of Entomology, Pennsylvania State University, State College, PA USA; 4 Department of Earth System Science, Tsinghua University, Beijing, China; 5 College of Management and Economics, Tianjin University, Tianjin, China; 6 Advanced Power and Energy Program, University of California, Irvine, Irvine, CA USA; 7 The World Bank, Washington, DC USA; 8 Centre for Ecological and Evolutionary Synthesis, Department of Biosciences, Faculty of Mathematics and Natural Sciences, University of Oslo, Oslo, Norway; 9 The Bartlett School of Sustainable Construction, University College London, London, UK; 10 Centre for Pandemics and One Health Research, Faculty of Medicine, University of Oslo, Oslo, Norway

**Keywords:** Interdisciplinary studies, Economics

Correction to: *Nature Communications* 10.1038/s41467-023-37075-x, published online 21 March 2023

In the Acknowledgements section of this article, the grant number relating to the National Natural Science Foundation of China given for Dabo Guan was incorrectly given as 7224100119 and should have been 72242105. The original article has been corrected.

