# Supplementary material for: Supply chains create global benefits from improved vaccine accessibility
Source: Nat Commun. 2023 Mar 21;14:1569. doi: 10.1038/s41467-023-37075-x (PMC10030081; doi:10.1038/s41467-023-37075-x)
Supplement: Supplementary file 1 — Supplementary Information [file 41467_2023_37075_MOESM1_ESM.pdf]

Supplementary information for

Supply chains create global benefits from improved vaccine accessibility

Daoping Wang[1,2], Ottar N. Bjørnstad[3], Tianyang Lei[4], Yida Sun[4], Jingwen Huo[4], Qi Hao[4], Zhao Zeng[5], Shupeng Zhu[6], Stéphane Hallegatte[7], Ruiyun Li[8], Dabo Guan[4,9,*], Nils C. Stenseth[8,10,*]

[1] Department of Computer Science and Technology, University of Cambridge, Cambridge, UK.

[2] The World Economic Forum, Geneva, Switzerland.

[3] Center for Infectious Disease Dynamics, Department of Entomology, Pennsylvania State University, PA, USA.

[4] Department of Earth System Science, Tsinghua University, Beijing, China.

[5] College of Management and Economics, Tianjin University, Tianjin, China.

[6] Advanced Power and Energy Program, University of California, Irvine, Irvine, CA, USA.

[7] The World Bank, Washington, DC, USA.

[8] Centre for Ecological and Evolutionary Synthesis, Department of Biosciences, University of Oslo, Oslo, Norway.

[9] The Bartlett School of Sustainable Construction, University College London, London, UK.

[10] Center for Pandemic and One Health Research, Sustainable Health Unit (SUSTAINIT), Faculty of Medicine, University of Oslo, Oslo, Norway

* Corresponding author.

Email: guandabo@tsinghua.edu.cn (D.G.); n.c.stenseth@mn.uio.no (N.C.S).

Supplementary Figures

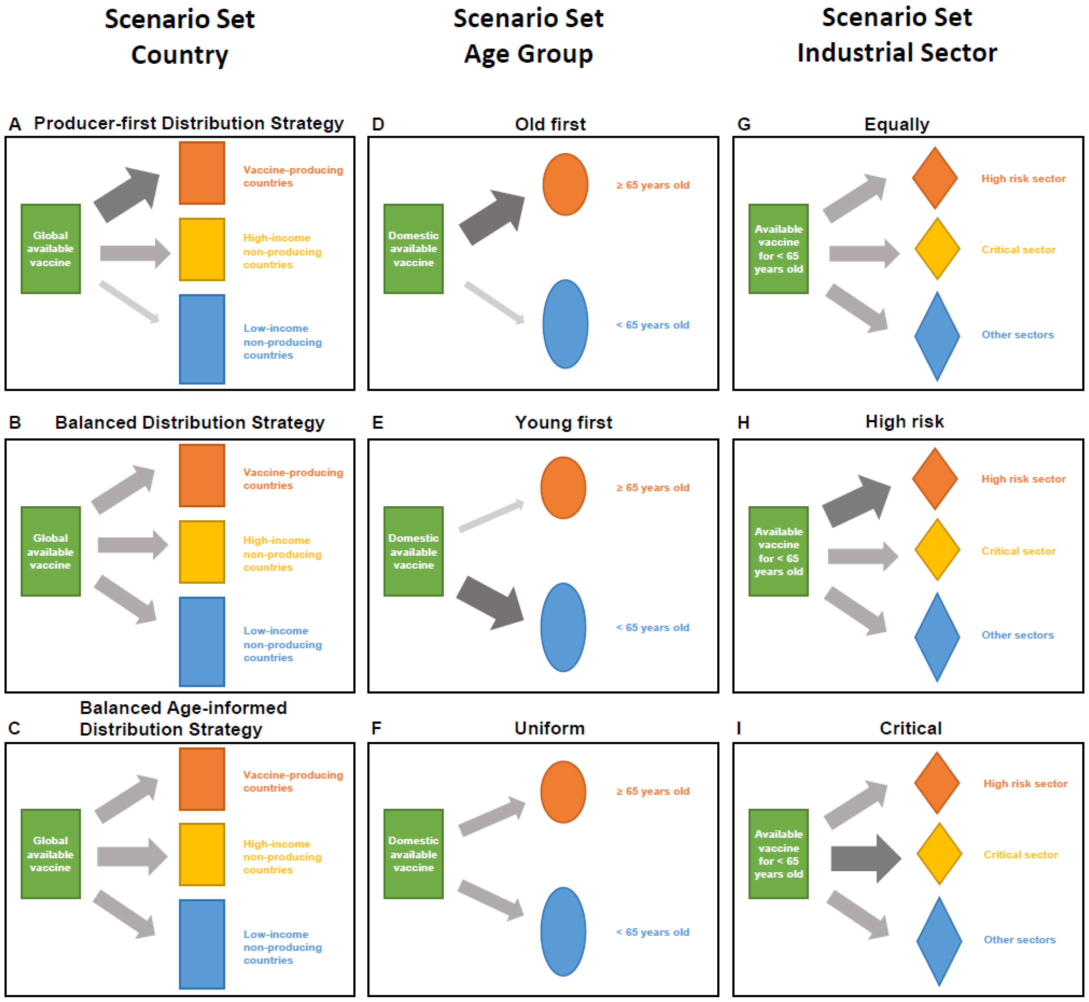

Supplementary Figure 1. Schematic diagram of the scenario sets.

Scenario C represents to what extent the vaccine-exporting country is willing to share the vaccine with other countries (specifically, a Producer-first Distribution Strategy vs two Balanced Distribution Strategies). Scenario A defines the allocation of the received vaccines within destination countries by age group. And scenario S defines the allocation of the received vaccines within young groups in destination countries by industrial sectors.

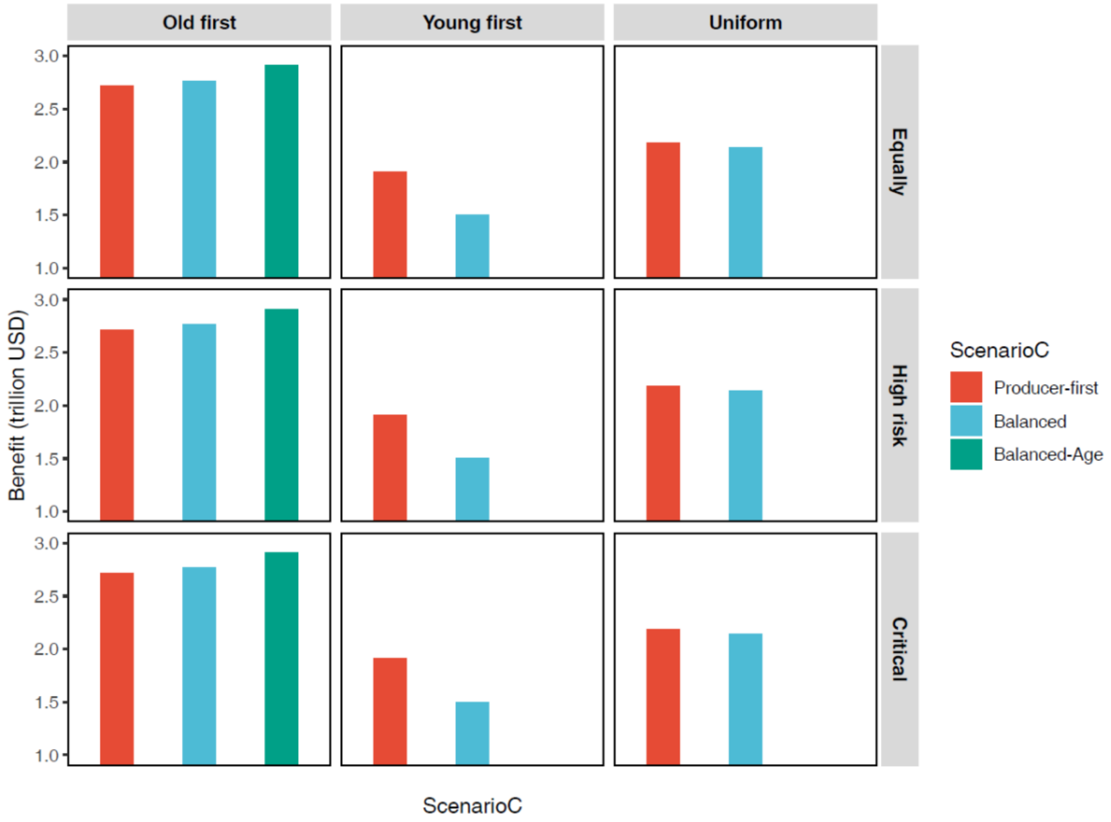

Supplementary Figure 2. Global health gains under in 21 scenarios.

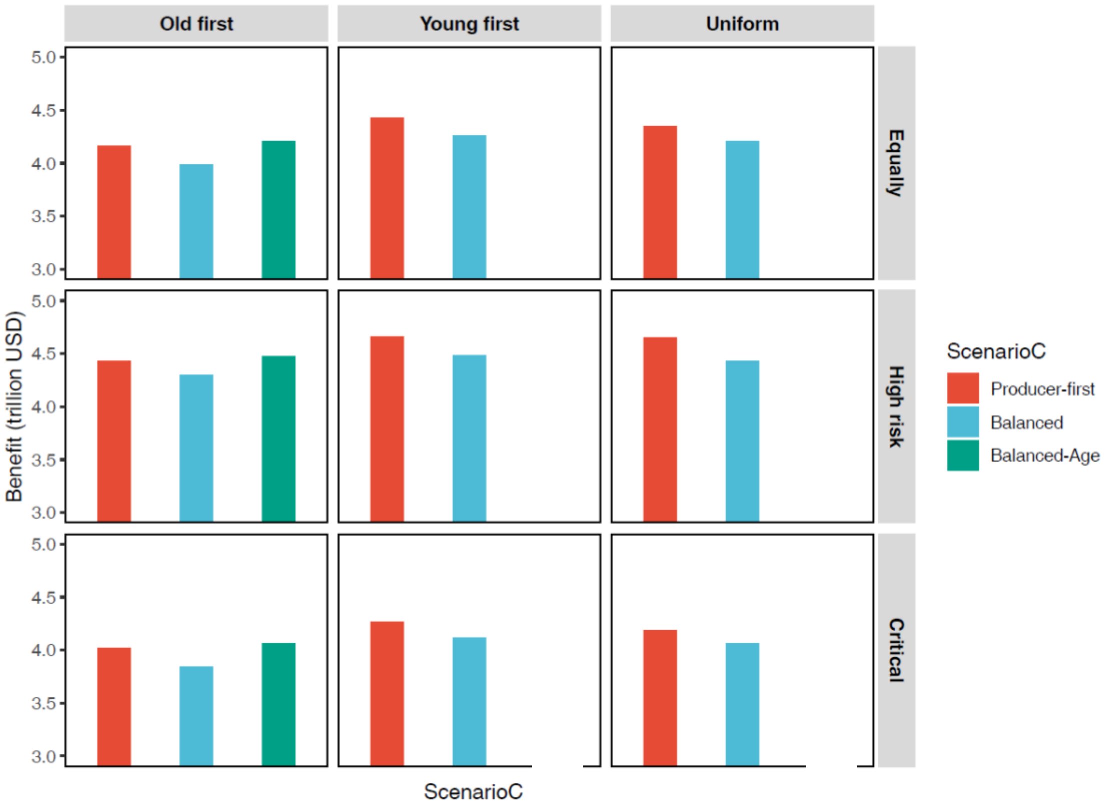

Supplementary Figure 3. Global lockdown-easing effect under in 21 scenarios.

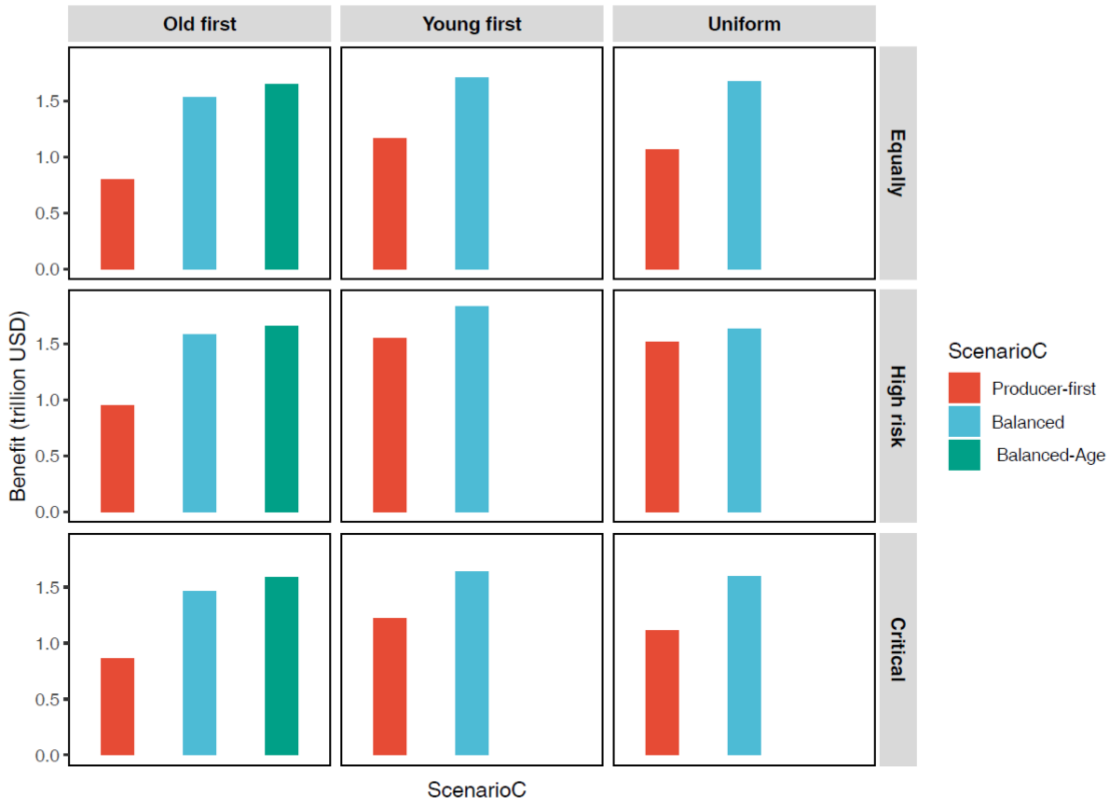

Supplementary Figure 4. Global supply-chain rebuilding benefit under in 21 scenarios.

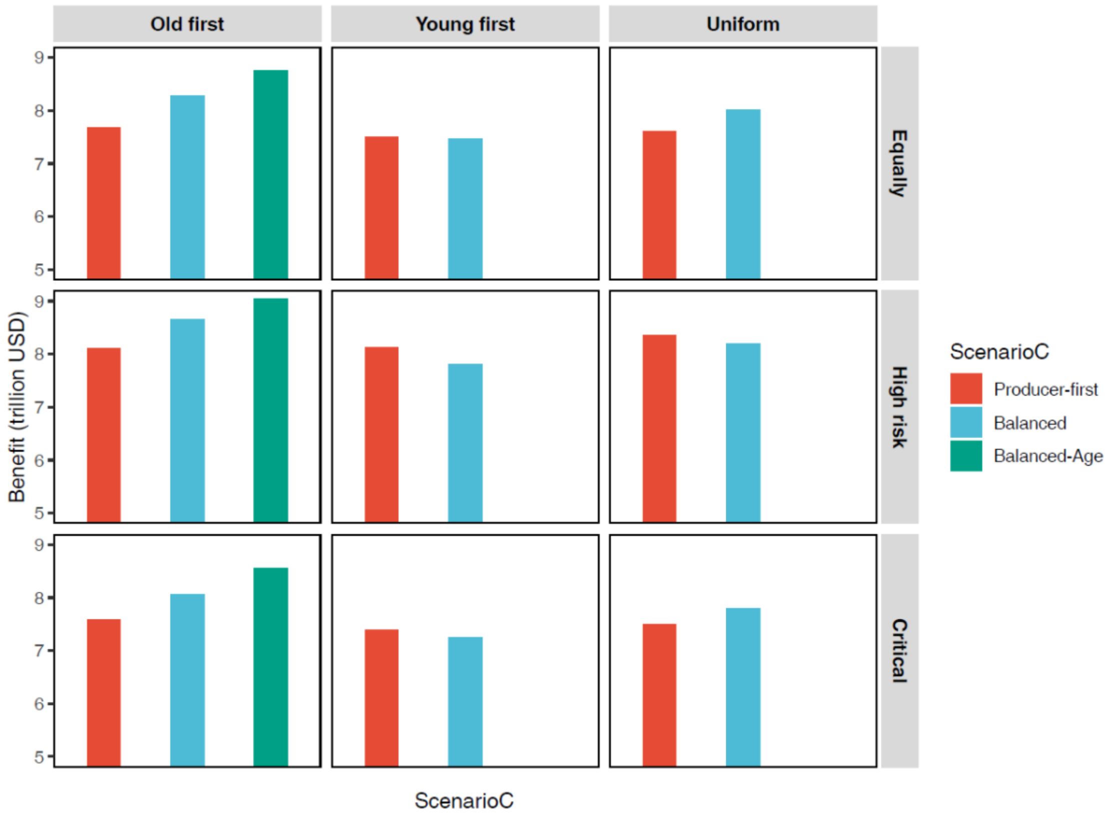

Supplementary Figure 5. Global benefits under in 21 scenarios.

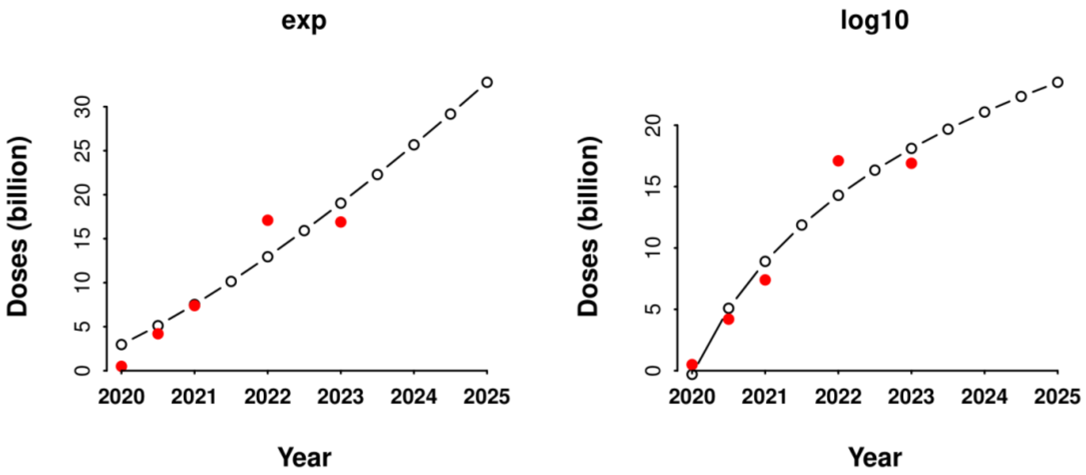

Supplementary Figure 6. Fitting results of vaccine production capacity.

It can be seen from the figure that the logarithmic function (right-hand side) fits better than exponential function form (lift-hand side). It is worth mentioning that there are too few sample points for fitting. If the estimated production capacity in 2024 and 2025 is too large or too small, it will have a certain degree of impact on the results of this study. We conducted +25% and -25% sensitivity tests, and find that the conclusions in the article will not change.

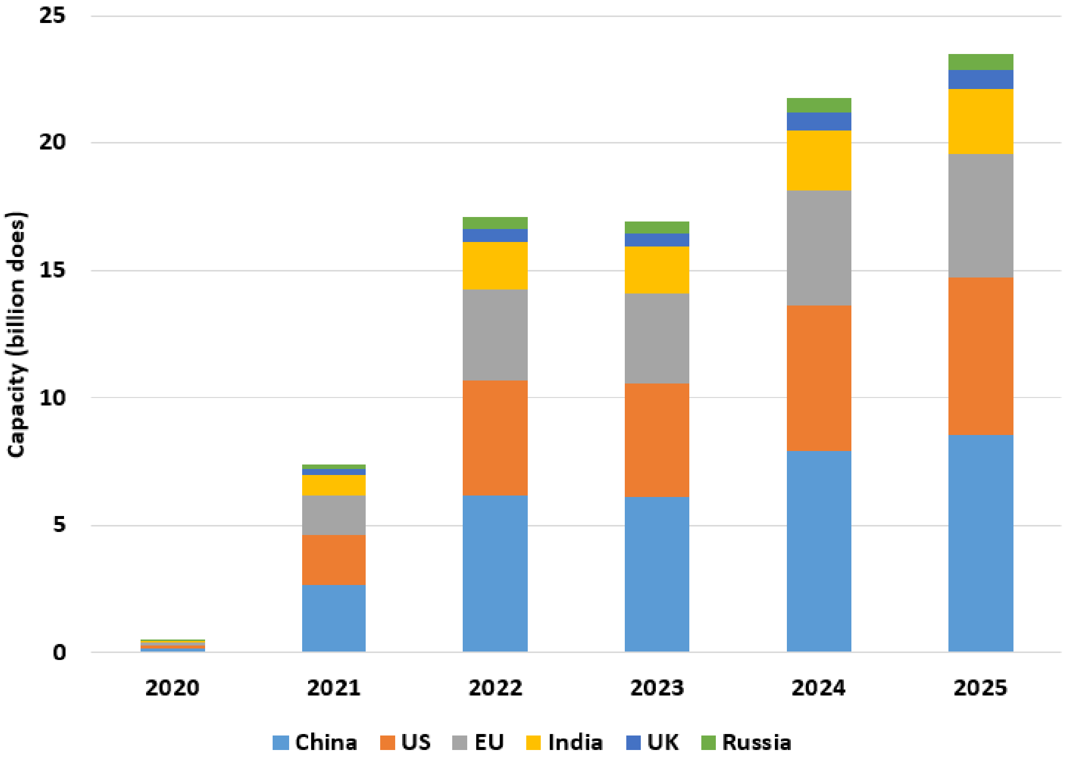

Supplementary Figure 7. Projected vaccine production capacity in six regions.

The data for 2020 to 2023 are from UNICEF[1], while the data for 2024 and 2025 are from estimates by the authors.

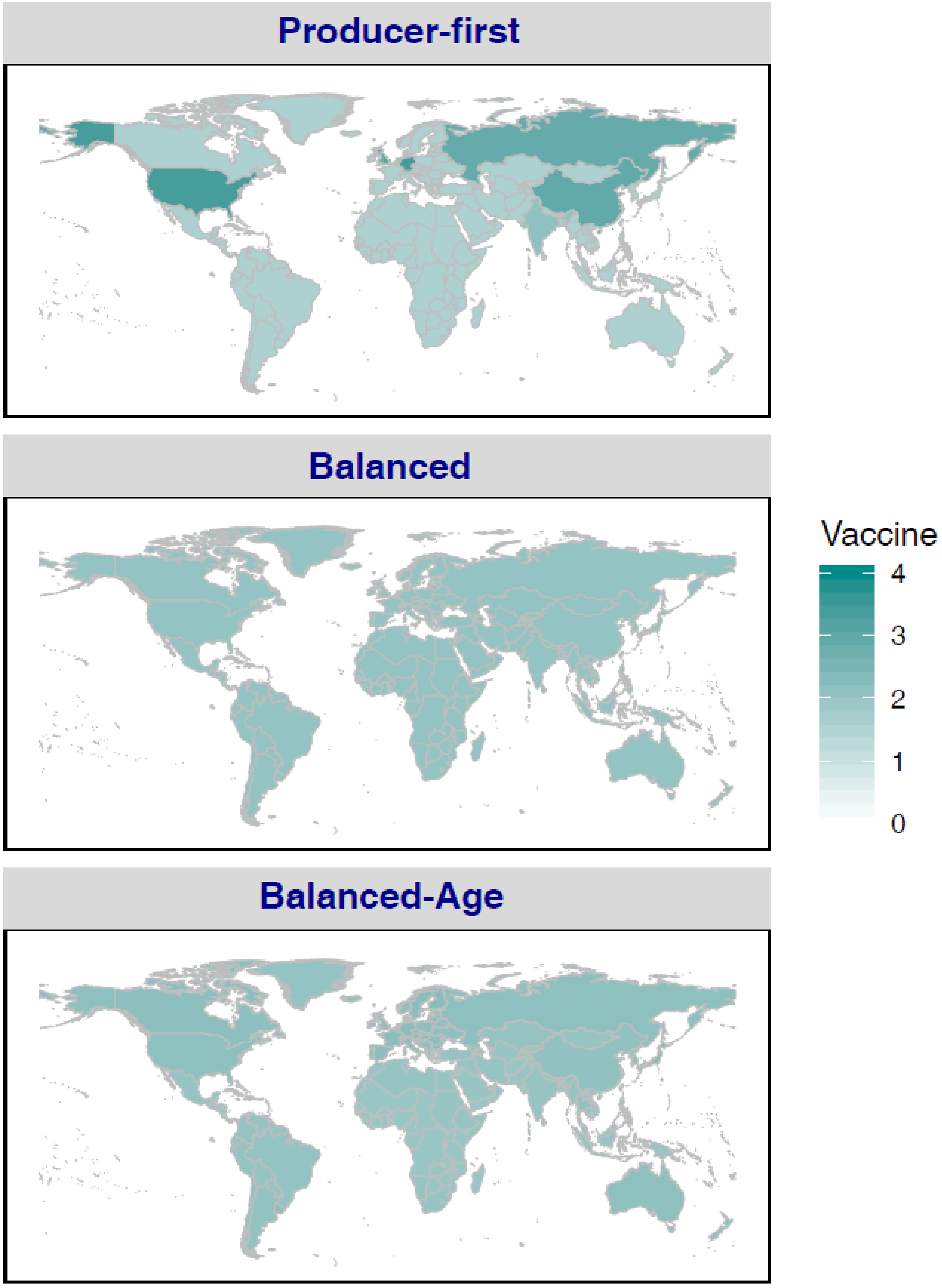

Supplementary Figure 8. The average doses of vaccine available per capita per year in each country/region during 2020-2025.

The panel on the top represents the number available under the Producer-first Distribution Strategy; the panel on the middle represents the number available under the Balanced Distribution Strategy; and the panel on the bottom represents the number available under the Balanced Age-informed Distribution Strategy.

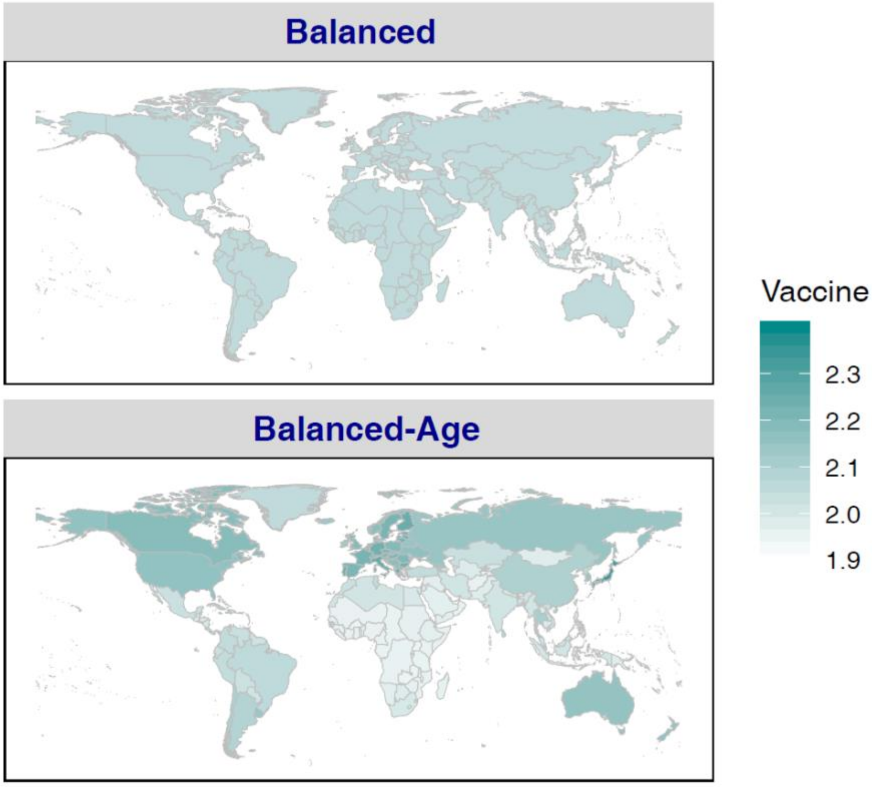

Supplementary Figure 9. The average doses of vaccine available per capita per year in each country/region during 2020-2025. (A detailed display of Fig. S8)

The panel on the top represents the number available under the Balanced Distribution Strategy, while the panel on the bottom represents the number available under the Balanced Age-informed Distribution Strategy.

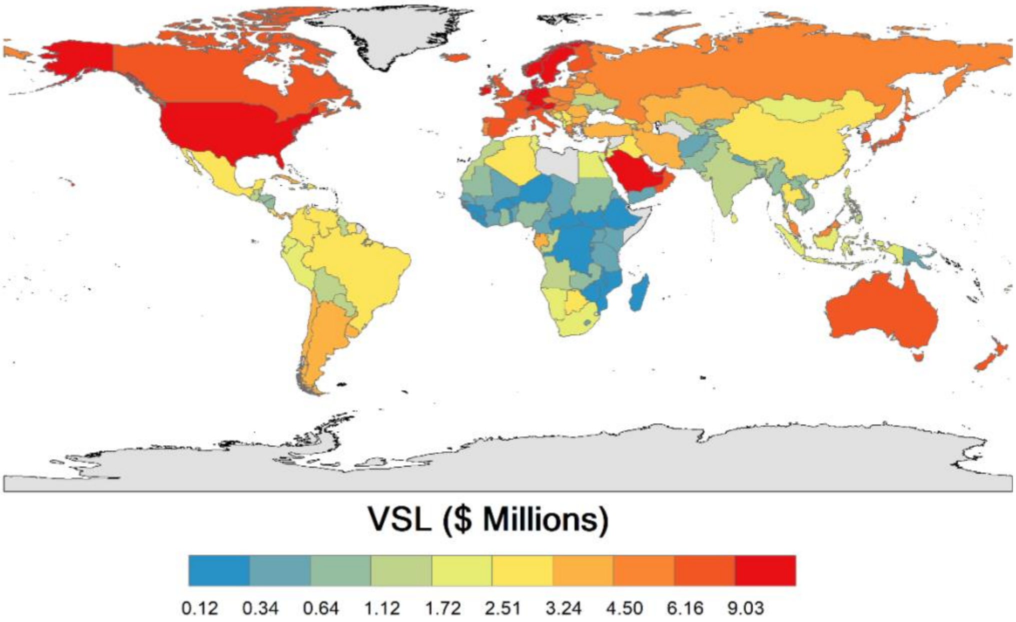

Supplementary Figure 10. Estimated VSL for 175 countries in 2019$.

The country based VSL estimation used in this research is adopted from the COVID-19 global health risks pricing study by Viscusi (Table 6)[2]. The estimation is based on the estimated VSL in the U.S. (11 million in 2019 US dollor), and coupled with an income elasticity of 1.0 to adjust the VSL to other countries using the fixed effects specification[3].

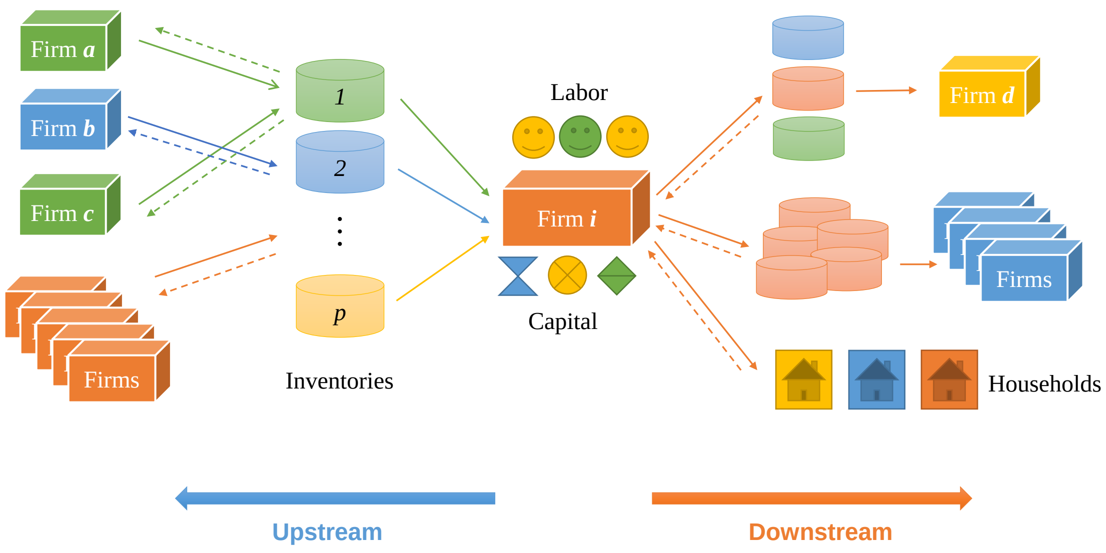

Supplementary Figure 11. Overview of the backbone of the recursive dynamic disaster impact assessment model.

Products flow from left to right (solid line), whereas orders flow in the opposite direction (dotted line). In each time step, firm *i* use the primary inputs (e.g., labour and capital) and inventories (i.e., intermediates) to produce a specific good, to meet the orders from its downstream clients (i.e., other firms and households). At the same time, firm *i* will issue orders to its upstream suppliers to restore its inventories, which is used in the production process, to a target level. In our model, some inventories of firm *i* may come from different firms (in practice, in the MRIO table for example, this refers to the same sector sourced from different regions). When one type of inventory has multiple suppliers, firm *i* will consider the order proportion of the equilibrium period and the current production capacity of each supplier when releases orders to them (the second improvement of our disaster footprint model on the ARIO model).

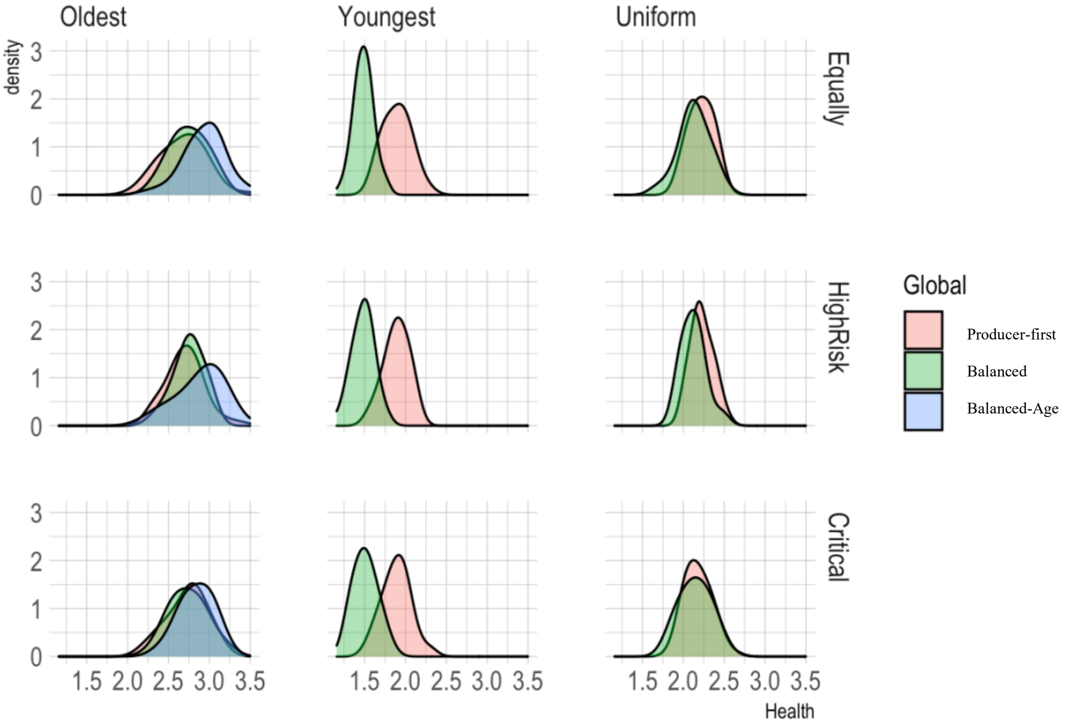

Supplementary Figure 12 | Distribution of health gains under different scenarios

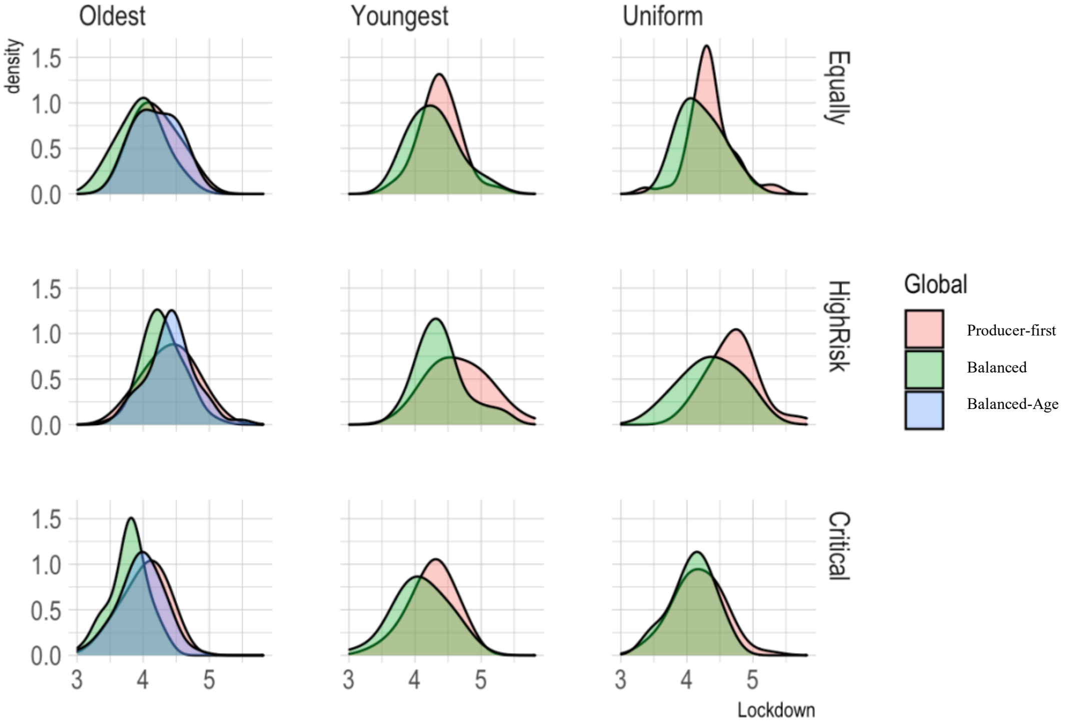

Supplementary Figure 13 | Distribution of lockdown-easing effect under different scenarios

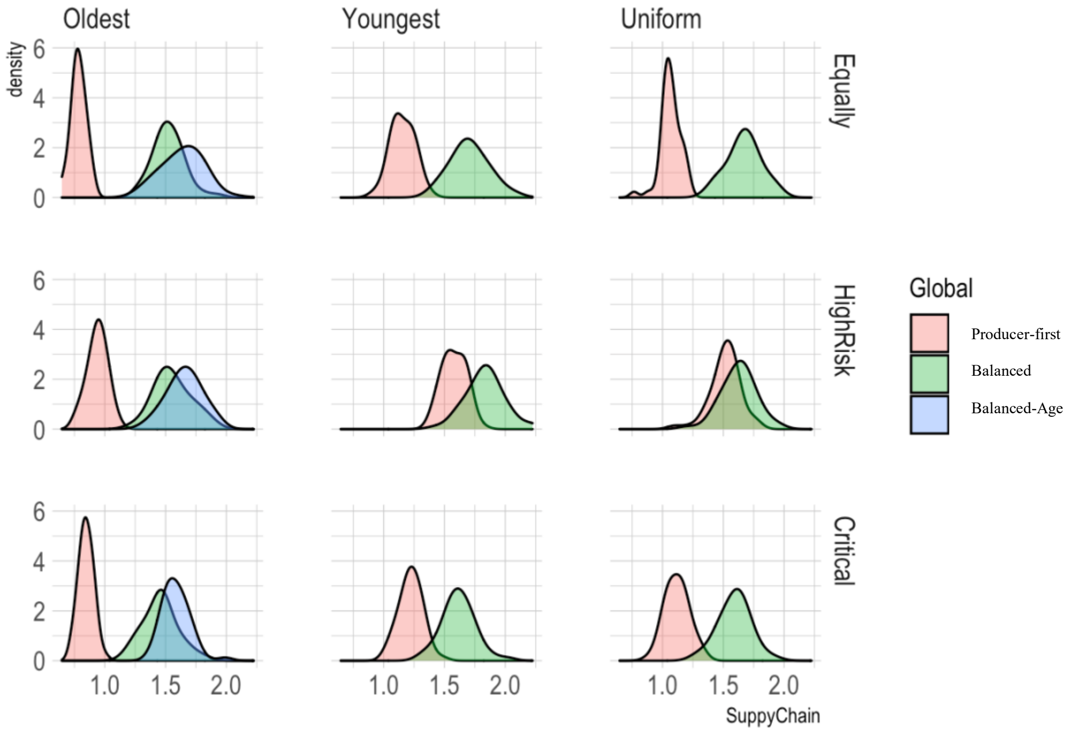

Supplementary Figure 14 | Distribution of supply-chain rebuilding benefit under different scenarios

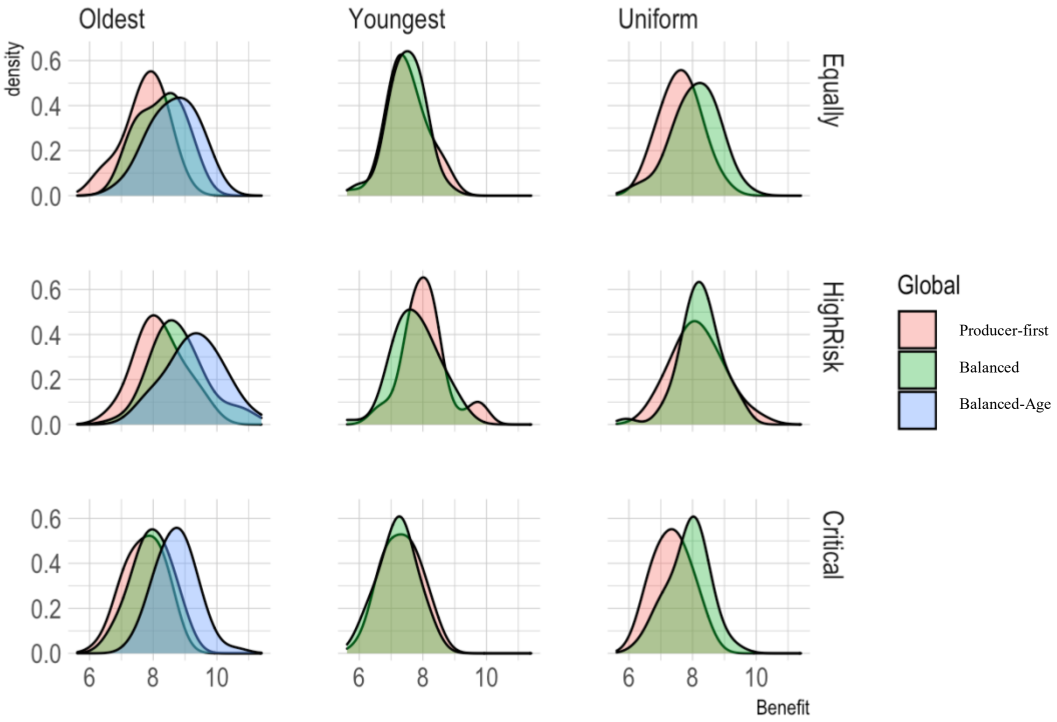

Supplementary Figure 15 | Distribution of global total under different scenarios

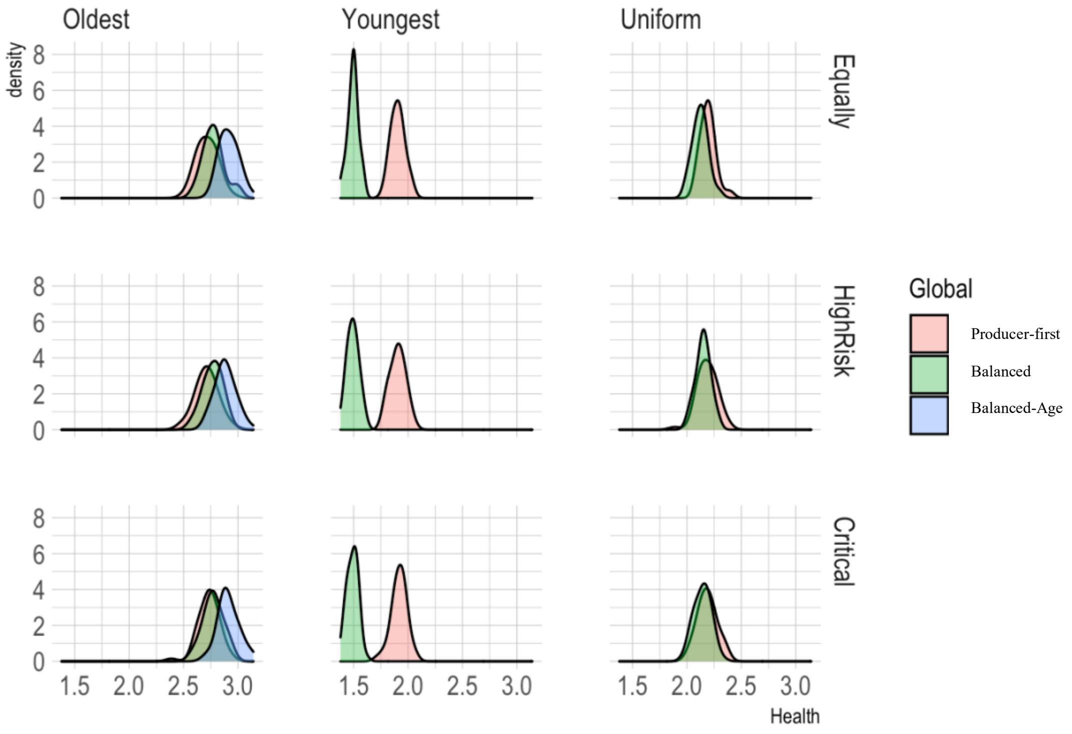

Supplementary Figure 16 | Distribution of health gains under different scenarios

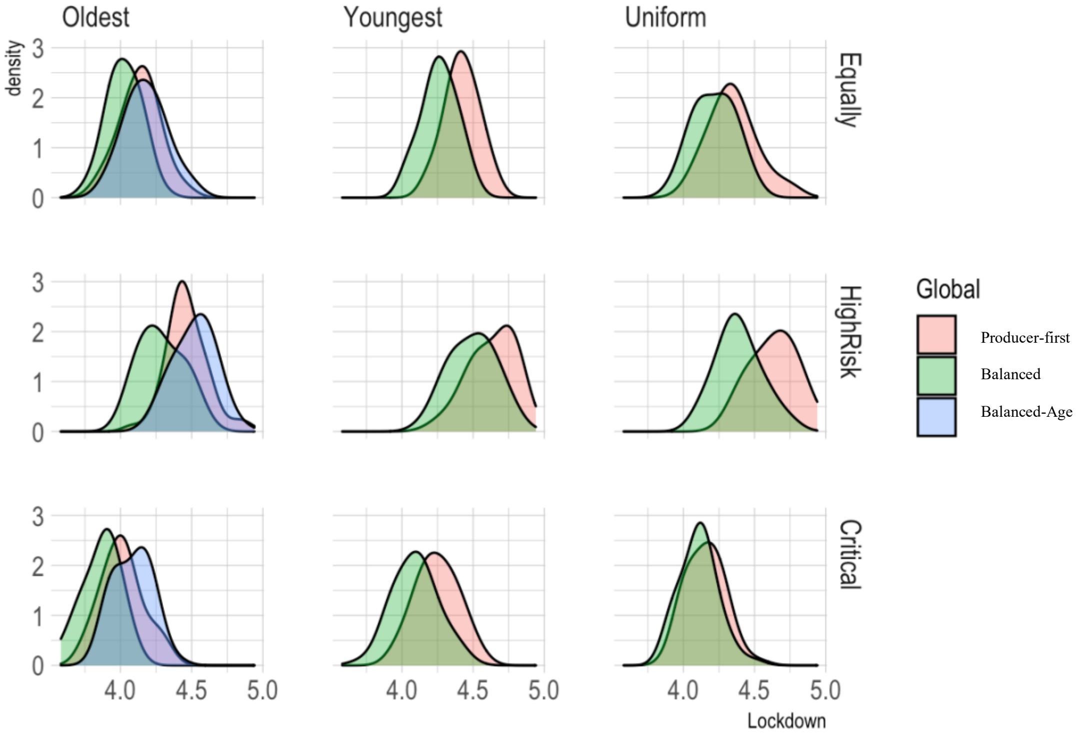

Supplementary Figure 17 | Distribution of lockdown-easing effect under different scenarios

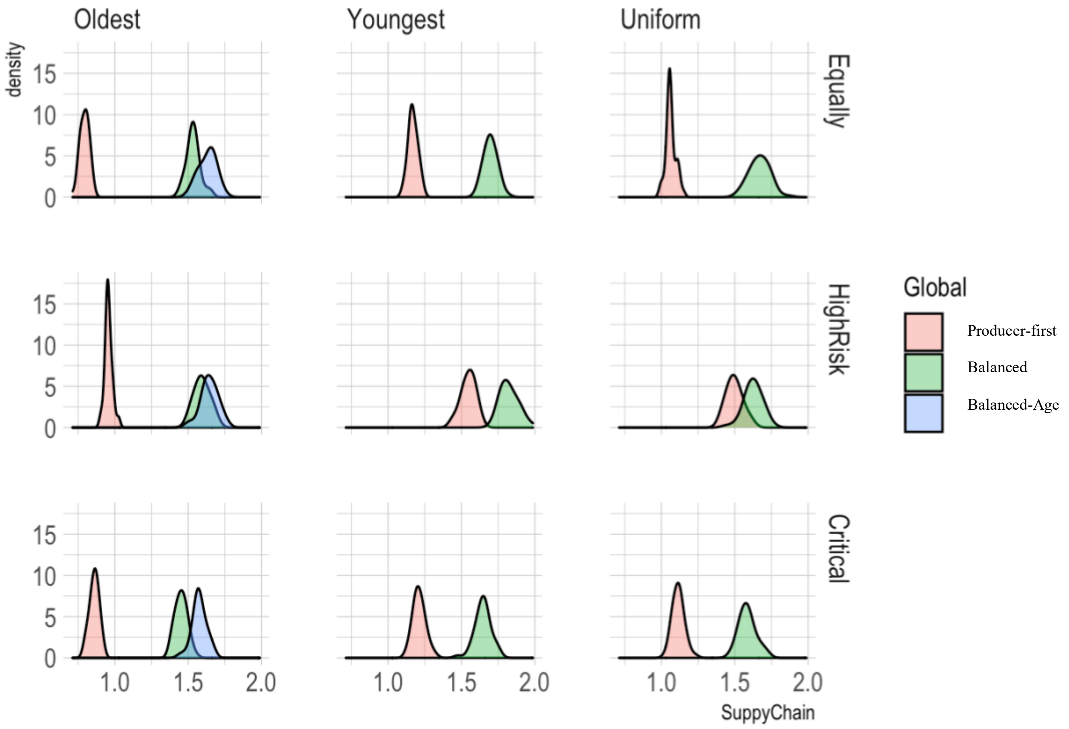

Supplementary Figure 18 | Distribution of supply-chain rebuilding benefit under different scenarios

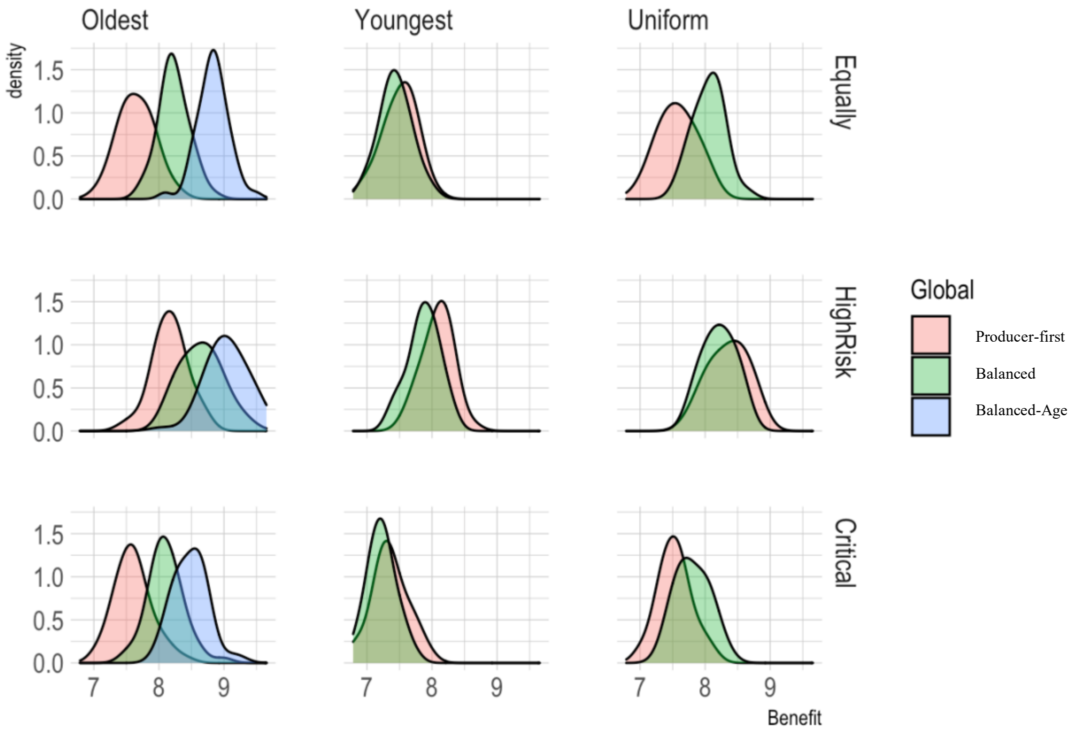

Supplementary Figure 19 | Distribution of global total under different scenarios

Supplementary Tables

Supplementary Table 1. Risk level of industrial sectors

No.	Sector code	Description	Risk level
1	pdr	Paddy rice	Low
2	wht	Wheat	Low
3	gro	Cereal grains nec	Low
4	v_f	Vegetables, fruit, nuts	Low
5	osd	Oil seeds	Low
6	c_b	Sugar cane, sugar beet	Low
7	pfb	Plant-based fibers	Low
8	ocr	Crops nec	Low
9	ctl	Bovine cattle, sheep and goats	Low
10	oap	Animal products nec	Low
11	rmk	Raw milk	Low
12	wol	Wool, silk-worm cocoons	Low
13	frs	Forestry	Low
14	fsh	Fishing	Low
15	coa	Coal	Low
16	oil	Oil	Low
17	gas	Gas	Low
18	oxt	Minerals nec	Low
19	cmt	Bovine meat products	Low
20	omt	Meat products nec	Low
21	vol	Vegetable oils and fats	Low
22	mil	Dairy products	Low
23	pcr	Processed rice	Low
24	sgr	Sugar	Low
25	ofd	Food products nec	Low
26	b_t	Beverages and tobacco products	Low
27	tex	Textiles	High
28	wap	Wearing apparel	High
29	lea	Leather products	High
30	lum	Wood products	Middle
31	ppp	Paper products, publishing	Middle
32	p_c	Petroleum, coal products	Middle
33	chm	Chemical products	Middle
34	bph	Basic pharmaceutical products	Middle
35	rpp	Rubber and plastic products	Middle
36	nmm	Mineral products nec	Middle

37	i_s	Ferrous metals	Middle
38	nfm	Metals nec	Middle
39	fmp	Metal products	Middle
40	ele	Computer, electronic and optic	Middle
41	eeq	Electrical equipment	Middle
42	ome	Machinery and equipment nec	Middle
43	mvh	Motor vehicles and parts	Middle
44	otn	Transport equipment nec	Middle
45	omf	Manufactures nec	Middle
46	ely	Electricity	Middle
47	gdt	Gas manufacture, distribution	Middle
48	wtr	Water	Middle
49	cns	Construction	Middle
50	trd	Trade	High
51	afs	Accommodation, Food and servic	High
52	otp	Transport nec	High
53	wtp	Water transport	High
54	atp	Air transport	High
55	whs	Warehousing and support activi	High
56	cmn	Communication	Low
57	ofi	Financial services nec	Low
58	ins	Insurance	Low
59	rsa	Real estate activities	Low
60	obs	Business services nec	Low
61	ros	Recreational and other service	High
62	osg	Public Administration and defe	Low
63	edu	Education	High
64	hht	Human health and social work a	High
65	dwe	Dwellings	Low

Note: Please refer to [4-6] for more information about the risk classification.

Supplementary Table 2. The 141 modeled regions and their demographic characteristics (2020).

RID	id	name	population	pop65+
1	aus	Australia	25687041	4164655
2	nzl	New Zealand	5084300	832214
3	xoc	Rest of Oceania	12310386	523874
4	chn	China	1402112000	167808567
5	hkg	Hong Kong	7481800	1361446
6	jpn	Japan	125836021	35733999
7	kor	Korea Republic of	51780579	8176623
8	mng	Mongolia	3278292	141370
9	twn	Taiwan	23694089	2207815
10	xea	Rest of East Asia	26428157	2487718
11	brn	Brunei Darussalam	437483	24368
12	khm	Cambodia	16718971	811346
13	idn	Indonesia	273523621	17129453
14	lao	Lao Peoples Democratic Republic	7275556	309879
15	mys	Malaysia	32365998	2325071
16	phl	Philippines	109581085	6039724
17	sgp	Singapore	5685807	759143
18	tha	Thailand	69799978	9044498
19	vnm	Viet Nam	97338583	7656664
20	xse	Rest of Southeast Asia	55728236	3449513
21	bgd	Bangladesh	164689383	8608320
22	ind	India	1380004385	90719952
23	npl	Nepal	29136808	1698136
24	pak	Pakistan	220892331	9605834
25	lka	Sri Lanka	21919000	2462130
26	xsa	Rest of South Asia	40240495	1098539
27	can	Canada	38005238	6880606
28	usa	United States of America	329484123	54796260
29	mex	Mexico	128932753	9822231
30	xna	Rest of North America	120270	11206
31	arg	Argentina	45376763	5157755
32	bol	Bolivia	11673029	873984
33	bra	Brazil	212559409	20389282
34	chl	Chile	19116209	2340332
35	col	Colombia	50882884	4610275
36	ecu	Ecuador	17643060	1339301
37	pry	Paraguay	7132530	485795
38	per	Peru	32971846	2876880

39	ury	Uruguay	3473727	524105
40	ven	Venezuela	28435943	2267112
41	xsm	Rest of South America	1373193	96911
42	cri	Costa Rica	5094114	522305
43	gtm	Guatemala	16858333	850004
44	hnd	Honduras	9904608	492576
45	nic	Nicaragua	6624554	376109
46	pan	Panama	4314768	368429
47	slv	El Salvador	6486201	561118
48	xca	Rest of Central America	397621	19915
49	dom	Dominican Republic	10847904	816598
50	jam	Jamaica	2961161	268807
51	pri	Puerto Rico	3194034	665233
52	tto	Trinidad and Tobago	1399491	161146
53	xcb	Caribbean	24428517	2584149
54	aut	Austria	8917205	1712460
55	bel	Belgium	11555997	2225019
56	bgr	Bulgaria	6927288	1487145
57	hrv	Croatia	4047200	860120
58	cyp	Cyprus	1207361	173966
59	cze	Czech Republic	10698896	2155078
60	dnk	Denmark	5831404	1175844
61	est	Estonia	1331057	271179
62	fin	Finland	5530719	1247425
63	fra	France	67391582	13986082
64	deu	Germany	83240525	18052747
65	grc	Greece	10715549	2387895
66	hun	Hungary	9749763	1965855
67	irl	Ireland	4994724	728128
68	ita	Italy	59554023	13877215
69	lva	Latvia	1901548	393381
70	ltu	Lithuania	2794700	576211
71	lux	Luxembourg	632275	90989
72	mlt	Malta	525285	112011
73	nld	Netherlands	17441139	3494301
74	pol	Poland	37950802	7111772
75	prt	Portugal	10305564	2346811
76	rou	Romania	19286123	3708898
77	svk	Slovakia	5458827	911577
78	svn	Slovenia	2100126	435519
79	esp	Spain	47351567	9459712

80	swe	Sweden	10353442	2104731
81	gbr	United Kingdom	67215293	12537902
82	che	Switzerland	8636896	1649435
83	nor	Norway	5379475	942838
84	xef	Rest of EFTA	404562	60797
85	alb	Albania	2837743	417278
86	blr	Belarus	9398861	1464361
87	rus	Russian Federation	144104080	22348998
88	ukr	Ukraine	44134693	7480452
89	xee	Rest of Eastern Europe	2617820	327045
90	xer	Rest of Europe	13212172	2333680
91	kaz	Kazakhstan	18754440	1482126
92	kgz	Kyrgyzstan	6591600	311644
93	tjk	Tajikistan	9537642	303274
94	xsu	Rest of Former Soviet Union	40263237	1926892
95	arm	Armenia	2963234	349747
96	aze	Azerbaijan	10110116	681586
97	geo	Georgia	3714000	566508
98	bhr	Bahrain	1701583	45112
99	irn	Iran Islamic Republic of	83992953	5513595
100	isr	Israel	9216900	1144218
101	jor	Jordan	10203140	403401
102	kwt	Kuwait	4270563	129646
103	omn	Oman	5106622	128228
104	qat	Qatar	2881060	48665
105	sau	Saudi Arabia	34813867	1217949
106	tur	Turkey	84339067	7574547
107	are	United Arab Emirates	9890400	125055
108	xws	Rest of Western Asia	99177839	3781731
109	egy	Egypt	102334403	5456144
110	mar	Morocco	36910558	2807655
111	tun	Tunisia	11818618	1048660
112	xnf	Rest of North Africa	50722330	3267826
113	ben	Benin	12123198	397455
114	bfa	Burkina Faso	20903278	503984
115	cmr	Cameroon	26545864	721027
116	civ	Cote dIvoire	26378275	760372
117	gha	Ghana	31072945	975799
118	gin	Guinea	13132792	387722
119	nga	Nigeria	206139587	5644234
120	sen	Senegal	16743930	520426

121	tgo	Togo	8278737	240499
122	xwf	Rest of Western Africa	67082442	1823758
123	xcf	Central Africa	30621589	816553
124	xac	South Central Africa	122427672	3423816
125	eth	Ethiopia	114963583	4066140
126	ken	Kenya	53771300	1349222
127	mdg	Madagascar	27691019	858614
128	mwi	Malawi	19129955	505326
129	mus	Mauritius	1265740	158472
130	moz	Mozambique	31255435	894476
131	rwa	Rwanda	12952209	404019
132	tza	Tanzania United Republic of	59734213	1579127
133	uga	Uganda	45741000	908279
134	zmb	Zambia	18383956	391950
135	zwe	Zimbabwe	14862927	447995
136	xec	Rest of Eastern Africa	73589328	2436785
137	bwa	Botswana	2351625	106110
138	nam	Namibia	2540916	91113
139	zaf	South Africa	59308690	3267576
140	xsc	Rest of South African Customs Union	3302416	152566
141	xtw	Rest of the World	7983	744

Data source:

https://www.gtap.agecon.purdue.edu/databases/regions.aspx?version=10.211#:~:text=GTAP%20Data%20Bases%3A%20GTAP%2010%20Data%20Base%20Final,%20%20China%20%2082%20more%20rows%20

https://data.worldbank.org/indicator/SP.POP.TOTL

https://data.worldbank.org/indicator/SP.POP.65UP.TO.ZS

Supplementary Table 3. GTAP sector classification and multiplier.

SID	Code	Name	Short Name	Multiplier	Rational
1	pdr	Paddy rice	Rice	0.5	
2	wht	Wheat	Wheat	0.5	
3	gro	Cereal grains nec	Cereals	0.5	
4	v_f	Vegetables, fruit, nuts	Veg & fruits	0.5	
5	osd	Oil seeds	Oilseed	0.5	
6	c_b	Sugar cane, sugar beet	Sugar crops	0.5	
7	pfb	Plant-based fibers	Fibre crops	0.5	1)Lower exposure risk of agricultural production activities. 2) Necessity
8	ocr	Crops nec	Crops nec	0.5	
9	ctl	Cattle, sheep and goats, horses	Livestock	0.5	
10	oap	Animal products nec	Poultry	0.5	
11	rmk	Raw milk	Milk	0.5	
12	wol	Wool, silk-worm cocoons	Animal fiber	0.5	
13	frs	Forestry	Forestry	0.5	
14	fsh	Fishing	Fishing	0.5	
15	coa	Coal	Coal	1	
16	oil	Oil	Oil	1	
17	gas	Gas	Gas	1	
18	oxt	Other Extraction	Other extraction	1	
19	cmt	Meat: cattle, sheep, goats, horse	Red meat	1	
20	omt	Meat products nec	White meat	1	
21	vol	Vegetable oils and fats	Oil	1	
22	mil	Dairy products	Dairy	1	
23	pcr	Processed rice	Rice products	1	
24	sgr	Sugar	Sugar	1	
25	ofd	Food products nec	Food	1	
26	b_t	Beverages and tobacco products	Drinks & tobacco	1	Ordinary setor
27	tex	Textiles	Textiles	1	
28	wap	Wearing apparel	Garments	1	
29	lea	Leather products	Leather	1	
30	lum	Wood products	Timber	1	
31	ppp	Paper products, publishing	Paper	1	
32	p_c	Petroleum, coal products	Petroleum	1	
33	chm	Chemical products	Chemicals	1	
34	bph	Basic pharmaceutical products	Medicament	1	
35	rpp	Rubber and plastic products	Rubber & Plastic	1	
36	nmm	Mineral products nec	Minerals	1	
37	i_s	Ferrous metals	Ferrous	1	
38	nfm	Metals nec	Metals	1	

39	fmp	Metal products	Metal products	1	
40	ele	Computer, electronic and optical products	Electronics	1	
41	eeq	Electrical equipment	Electrical	1	
42	ome	Machinery and equipment nec	Machinnery	1	
43	mvh	Motor vehicles and parts	Motor parts	1	
44	otn	Transport equipment nec	Trans. equip	1	
45	omf	Manufactures nec	Other manuf.	1	
46	ely	Electricity	Electricity	0.1	Lifeline
47	gdt	Gas manufacture, distribution	Gas	0.1	
48	wtr	Water	Water	0.1	
49	cns	Construction	Construction	1	Ordinary setor
50	trd	Trade	Trade	1	
51	afs	Accommodation, Food and service activities	Catering	1	
52	otp	Transport nec	Land transport	1	
53	wtp	Water transport	Water transport	1	
54	atp	Air transport	Air transport	1	
55	whs	Warehousing and support activities	Warehousing	1	
56	cmn	Communication	Communication	0.1	Work on line
57	ofi	Financial services nec	Finance	0.1	
58	ins	Insurance	Insurance	0.1	
59	rsa	Real estate activities	Real estate	0.1	
60	obs	Business services nec	Business	0.1	
61	ros	Recreational and other services	Tourism	1	
62	osg	Public Administration and defense	Administration	0.1	Special setor
63	edu	Education	Education	0.1	Work on line
64	hht	Human health and social work activities	Public health	0	Special setor
65	dwe	Dwellings	Dwellings	1	Ordinary setor

Note: GTAP database classifies agriculture, food, resource extraction, manufacturing, and service activities (65 sectors in total) to describe all economic activities in each country/region. COVID-19 lockdown measures have different effects on labor supply in different sectors. We set a specific multiplier for each sector based on three factors, i.e., the exposure level of the sector's work, whether it is the lifeline, and whether it is possible to work at home. If a sector's work exposure level is low, or it is the lifeline sector, or it is easy to work at home, its' multiplier will be small, vice versa.

Supplementary Table 4 Sector aggregation scheme

No	Code	Description	ID	Code	Sector Name
1	pdr	Paddy rice	1	GrainsCrops	Grains and Crops
2	wht	Wheat	1	GrainsCrops	Grains and Crops
3	gro	Cereal grains nec	1	GrainsCrops	Grains and Crops
4	v_f	Vegetables, fruit, nuts	1	GrainsCrops	Grains and Crops
5	osd	Oil seeds	1	GrainsCrops	Grains and Crops
6	c_b	Sugar cane, sugar beet	1	GrainsCrops	Grains and Crops
7	pfb	Plant-based fibers	1	GrainsCrops	Grains and Crops
8	ocr	Crops nec	1	GrainsCrops	Grains and Crops
9	ctl	Bovine cattle, sheep and goats	2	MeatLstk	Meat and Milk
10	oap	Animal products nec	2	MeatLstk	Meat and Milk
11	rmk	Raw milk	2	MeatLstk	Meat and Milk
12	wol	Wool, silk-worm cocoons	2	MeatLstk	Meat and Milk
13	frs	Forestry	3	Extraction	Extraction
14	fsh	Fishing	3	Extraction	Extraction
15	coa	Coal	3	Extraction	Extraction
16	oil	Oil	3	Extraction	Extraction
17	gas	Gas	3	Extraction	Extraction
18	oxt	Minerals nec	3	Extraction	Extraction
19	cmt	Bovine meat products	2	MeatLstk	Meat and Milk
20	omt	Meat products nec	2	MeatLstk	Meat and Milk
21	vol	Vegetable oils and fats	4	ProcFood	Food Manufacturing
22	mil	Dairy products	4	ProcFood	Food Manufacturing
23	pcr	Processed rice	1	GrainsCrops	Grains and Crops
24	sgr	Sugar	4	ProcFood	Food Manufacturing
25	ofd	Food products nec	4	ProcFood	Food Manufacturing
26	b_t	Beverages and tobacco products	4	ProcFood	Food Manufacturing
27	tex	Textiles	5	TextWapp	Textiles
28	wap	Wearing apparel	5	TextWapp	Textiles
29	lea	Leather products	6	LightMnfc	Light Manufacturing
30	lum	Wood products	6	LightMnfc	Light Manufacturing

31	ppp	Paper products, publishing	6	LightMnfc	Light Manufacturing
32	p_c	Petroleum, coal products	7	HeavyMnfc	Heavy Manufacturing
33	chm	Chemical products	7	HeavyMnfc	Heavy Manufacturing
34	bph	Basic pharmaceutical products	7	HeavyMnfc	Heavy Manufacturing
35	rpp	Rubber and plastic products	7	HeavyMnfc	Heavy Manufacturing
36	nmm	Mineral products nec	7	HeavyMnfc	Heavy Manufacturing
37	i_s	Ferrous metals	7	HeavyMnfc	Heavy Manufacturing
38	nfm	Metals nec	7	HeavyMnfc	Heavy Manufacturing
39	fmp	Metal products	6	LightMnfc	Light Manufacturing
40	ele	Computer, electronic and optic	7	HeavyMnfc	Heavy Manufacturing
41	eeq	Electrical equipment	7	HeavyMnfc	Heavy Manufacturing
42	ome	Machinery and equipment nec	7	HeavyMnfc	Heavy Manufacturing
43	mvh	Motor vehicles and parts	6	LightMnfc	Light Manufacturing
44	otn	Transport equipment nec	6	LightMnfc	Light Manufacturing
45	omf	Manufactures nec	6	LightMnfc	Light Manufacturing
46	ely	Electricity	8	Util_Cons	Construction
47	gdt	Gas manufacture, distribution	8	Util_Cons	Construction
48	wtr	Water	8	Util_Cons	Construction
49	cns	Construction	8	Util_Cons	Construction
50	trd	Trade	9	TransComm	Transportation
51	afs	Accommodation, Food and servic	9	TransComm	Transportation
52	otp	Transport nec	9	TransComm	Transportation
53	wtp	Water transport	9	TransComm	Transportation
54	atp	Air transport	9	TransComm	Transportation

55	whs	Warehousing and support activi	9	TransComm	Transportation
56	cmn	Communication	9	TransComm	Transportation
57	ofi	Financial services nec	10	OthServices	Other Services
58	ins	Insurance	10	OthServices	Other Services
59	rsa	Real estate activities	10	OthServices	Other Services
60	obs	Business services nec	10	OthServices	Other Services
61	ros	Recreational and other service	10	OthServices	Other Services
62	osg	Public Administration and defe	10	OthServices	Other Services
63	edu	Education	10	OthServices	Other Services
64	hht	Human health and social work a	10	OthServices	Other Services
65	dwe	Dwellings	10	OthServices	Other Services

Supplementary Table 5. Total number of COVID-19 vaccine doses produced by country (March 03, 2021)

Country	doses	%
China	141624000	0.362453019
US	103000000	0.263604057
Germany/Belgium	70534055	0.180515175
India	42390000	0.108487145
UK	12200000	0.031223005
Netherlands/Belgium	10496982	0.026864534
Russia	10492500	0.026853064

Source: https://www.statista.com/chart/24492/total-covid-19-vaccine-production-by-country/

Supplementary Table 6. Parameters of epidemiological model

Parameter	Values	Details
Baseline reproduction number, R_0	3.5	Assumed
Average duration of infection, $1/\gamma$	7 days	Prem et al[8]
Immune duration, $1/\omega$	1 year	Assumed
Average rate of aging, a	1 for all age classes	Assumed
Infection-fatality ratio	i.e., age-specific IFR	Estimated from Davies et al[9]
Demography	i.e., age-specific proportion of population	Ali et al[10]
Social mixing pattern, C	i.e., age-structured number of contacts	United Nations Statistics Division [11]

Supplementary Table 7. Sensitivity analyses: allocation mode in the economic model

	Proportional rationing scheme				Mix of priority system and proportional rationing		
	Producer-first	Balanced	Balanced Age-informed		Producer-first	Balanced	Balanced Age-informed
Health gains	2.72	2.77	2.91		2.72	2.77	2.91
Lockdown-easing benefits	4.43	4.3	4.48		4.43	4.3	4.48
**Supply-chain rebuilding benefits**	**0.95**	**1.59**	**1.66**		**0.87**	**1.26**	**1.43**
**Total benefits**	**8.1**	**8.65**	**9.05**		**8.02**	**8.33**	**8.82**

Supplementary Table 8. Sensitivity analyses: demand structure

	Pre-COVID				Frozen tourism		
	Producer-first	Balanced	Balanced Age-informed		Producer-first	Balanced	Balanced Age-informed
Health gains	2.72	2.77	2.91		2.72	2.77	2.91
Lockdown-easing benefits	4.43	4.3	4.48		4.43	4.3	4.48
**Supply-chain rebuilding benefits**	**0.95**	**1.59**	**1.66**		**0.93**	**1.55**	**1.63**
**Total benefits**	**8.1**	**8.65**	**9.05**		**8.08**	**8.62**	**9.02**

Supplementary Text

Uncertainty from estimates of vaccine production capacities

The COVID-19 and vaccine manufacturing capabilities are changing rapidly. The vaccine production capacity pathway used in this study is only the best estimates when the study carried out and an application case of the proposed approach. Considering that COVID-19 and vaccine production capacity is still changing rapidly and the different starting points for future pandemics, here, we analyze uncertainties arising from estimates of vaccine production capacity using a Monte Carlo approach.

We assume that the vaccine production capacity of each vaccine-producing country takes a random value (uniform distribution) within ±25% based on its estimated production capacity. And then, we use these random combinations as the initial input to our model. Supplementary Figure 12-15 show the distribution of the three types of benefits and the distribution of total benefit, respectively. Distribution of global total benefit shifted right from "Producer-first Distribution Strategy" to "Balanced Distribution Strategy" scenarios under the default "Oldest" and "High Risk" scenario.

Uncertainty from model parameters

We assume that each parameter takes a random value (uniform distribution) within ±10% based on its value used in this study. And then, we use these random combinations as the parameter set to our model.

Supplementary Figure 16-19 show the distribution of the three types of benefits and the distribution of total benefit, respectively. Distribution of global total benefit shifted right from "Producer-first Distribution Strategy" to "Balanced Distribution Strategy" scenarios under the default "Oldest" and "High Risk" scenario.

Sensitivity analyses: allocation mode in the economic model

In order to analyse how different allocation modes affect the conclusions of this paper, we compare two allocation modes here. One is the proportional rationing scheme used in this study, and another is a mix of priority system and proportional rationing.

A proportional rationing scheme means a firm allocates its output in proportion to its orders. This is actually a reflection of the macro results under the market mechanism.

While in a mixed priority system and proportional rationing scheme, if a firm cannot satisfy total demand, its production goes first to its biggest client. And then, all other

clients are assumed equally rationed: what a client gets is proportional to what it ordered. This partly reflects the fact that firms tend to prioritize maintaining their key business links.

The simulation results under the two allocation modes are shown in Supplementary Table 7. we find that the conclusion in the manuscript remains under different allocation modes. In addition, a new finding is the mixed scheme is not as efficient as the proportional rationing scheme used in this study. That makes sense, because the marginal contribution of intermediates that were used to fulfil the demand of specific agents will become lower.

Sensitivity analyses: demand structure

In order to analyse how change of the demand structure affect the conclusions of this paper, we compare two scenarios here. One is the "pre-COVID" scenario used in this study, and another is a scenario in which global tourism is frozen ("Frozen tourism").

In the "Frozen tourism" scenario, we adjusted the demand for tourism (Recreational and other services in GTAP) in all countries around the world to zero in 2020. We assume that the demand will recover linearly over the next five years. Using tourism demand changes as an example, we test the sensitivity of the conclusions to changes in demand structure.

The simulation results under the two scenarios are shown in Supplementary Table 8. We find that the conclusion in the manuscript remains under different demand structure change scenarios, which reflects, to a certain extent, that our estimate is robust.

Current Vaccine Distribution

Current COVID-19 vaccination status varies greatly among countries worldwide, with a range of 0 to 123.2% variation in vaccination rates. The countries with the highest vaccination rates globally are Seychelles (123.2%) and Israel (120.9%), both with rates of 120 percent or higher. Besides, the UAE is another country with a vaccination rate exceeding 100%, which has reached 103.7%. Other countries whose vaccination rates are relatively high (>40%) are mostly concentrated in Europe and the Middle East, and also including three American countries, the United States (79.9%), Chile (73.9%), Uruguay (55.8%) and the small Asian country of Bhutan (62.2%). In general, these countries with high COVID-19 vaccination rates fall into three categories: developed countries with more advanced health care systems, high-income energy-exporting

countries, and developing economies with smaller populations.

Countries with moderate COVID-19 vaccination rates (10-40%) are mainly located in South America and East Asia, with Mexico and Russia, among others, also falling within this range. In contrast, vaccination rates in regions such as almost all of Africa, northwestern South America and Oceania are below 10%, that is very low. Moreover, the rates in less developed areas like Cameroon, Niger and South Sudan are even close to zero and can be ignored.

Current Supply Agreements

Although many countries presently have low COVID-19 vaccination rates, they have reached a series of supply agreements with vaccine manufacturers that have resulted in massive increases in population coverage when involving the number of vaccines in the agreements. There are currently a total of 43 countries with the number of vaccines in the agreements to achieve full population coverage. For example, Canada’s vaccination rate is 32.6% now, but with the agreements its coverage rate could reach a horrific 605.42%, equivalent to 6 doses of vaccine for every Canadian. Unlike the current distribution of vaccines, the countries with more agreements are absolute high-income countries, concentrated in Europe, North America and Oceania. However, the countries with less agreements are mostly low- and middle-income Asian countries such as Pakistan (1.03%), Georgia (1.25%), Iran (1.49%), and Uzbekistan (2.99%), whose current vaccination rates are also relatively low.

Current Price Information

Due to various factors, vaccine prices vary widely in different countries and groups. Currently, the most expensive vaccine in the world is available in Argentina, with a price of $40.00 per dose, which is Inactivated SARS-CoV-2 vaccine developed and manufactured by Beijing Institute of Biological Products (CNBG). On the contrary, the cheapest vaccine being sold is Covishield in India, developed by AstraZeneca and manufactured by Serum Institute of India, which costs only $2.06 per dose and just is 1/20 of the most expensive one. In summary, vaccine prices are currently higher in the more economically developed regions such as Argentina, the United States, the European Commission, and China, and the private markets of less developed countries like Thailand, Nepal, and Pakistan. Inversely, vaccine prices are relatively low in

regions such as India, African Union, Latin America, and the COVAX AMC implementation region.

The price of COVID-19 vaccine also has a clear relationship with the vaccine manufacturer. Beijing Institute of Biological Products (CNBG) manufactures the highest average price of vaccines at $31.09, with even the lowest priced vaccine sold in Senegal is priced at $18.60. In addition, vaccines manufactured by companied such as Moderna, Bharat Biotech, and Sinovac are also more expensive, which are used in high-income countries and private markets in less developed countries. Serum Institute of India produces a large number of vaccines at low prices, with an average of only $4.88. The company's most expensive vaccine, sold in the private market in Bangladesh, costs just $13.27, which is lower than most vaccine used currently. Other manufacturers such as AstraZeneca, Uniao Quimica Farmaceutica Nacional, and Fiocruz produce vaccines at relatively low prices, too.

Supplementary References

1 UNICEF. *COVID-19 Vaccine Market Dashboard*, <https://app.powerbi.com/view?r=eyJrIjoiNmE0YjZiNzUtZjk2OS00ZTg4LThlMzMtNTRhNzE0NzA4YmZlIiwidCI6Ijc3NDEwMTk1LTE0ZTEtNGZiOC05MDRiLWFiMTg5MjAyMzY2NyIsImMiOjh9&pageName=ReportSectiona329b3eafd86059a947b> (2021).

2 Viscusi, W. K. Pricing the global health risks of the COVID-19 pandemic. *Journal of Risk and Uncertainty* **61**, 101-128 (2020).

3 Viscusi, W. K. The role of publication selection bias in estimates of the value of a statistical life. *American Journal of Health Economics* **1**, 27-52 (2015).

4 Administration, O. S. a. H. *Hazard Recognition*, <https://www.osha.gov/coronavirus/hazards> (2021).

5 Administration, O. S. a. H. *Protecting Workers: Guidance on Mitigating and Preventing the Spread of COVID-19 in the Workplace*, <https://www.osha.gov/coronavirus/safework> (2021).

6 Hawkins, D. Differential occupational risk for COVID-19 and other infection exposure according to race and ethnicity. *American Journal of Industrial Medicine* **63**, 817-820, doi:10.1002/ajim.23145 (2020).

7 Guan, D. & Hallegatte, S. *The containment divide: COVID-19 lockdowns and basic needs in developing countries*, <https://blogs.worldbank.org/climatechange/containment-divide-covid-19-coronavirus-lockdowns-and-basic-needs-developing-countries> (2020).

8 Prem, K. *et al.* The effect of control strategies to reduce social mixing on outcomes of the COVID-19 epidemic in Wuhan, China: a modelling study. *The Lancet Public Health* **5**, e261-e270, doi:10.1016/s2468-2667(20)30073-6 (2020).

9 Davies, N. G. *et al.* Age-dependent effects in the transmission and control of COVID-19 epidemics. *Nature Medicine* **26**, 1205-1211, doi:10.1038/s41591-020-0962-9 (2020).

10 Ali, S. T. *et al.* Serial interval of SARS-CoV-2 was shortened over time by nonpharmaceutical interventions. *Science* **369**, 1106-1109, doi:10.1126/science.abc9004 (2020).

11 United Nations Statistics Division, Population by age, sex and urban/rural residence; http://unstats.un.org/unsd/default.htm.
