# Supplementary material for: Supply chains create global benefits from improved vaccine accessibility
Source: Nat Commun. 2023 Mar 21;14:1569. doi: 10.1038/s41467-023-37075-x (PMC10030081; doi:10.1038/s41467-023-37075-x)
Supplement: Supplementary file 3 — Description of Additional Supplementary Files [file 41467_2023_37075_MOESM3_ESM.pdf]

**Description of Additional Supplementary Files**

File Name: Supplementary Data 1
Description: Proportion of critical labour in each industrial sector.

File Name: Supplementary Data 2
Description: Health gains for each country under all combinations of different sets of scenarios.

File Name: Supplementary Data 3
Description: Lockdown-easing effect for each country under all combinations of different sets of scenarios.

File Name: Supplementary Data 4
Description: Supply-chain rebuilding benefit for each country under all combinations of different sets of scenarios.

File Name: Supplementary Data 5
Description: People saved for each country under all combinations of different sets of scenarios.

File Name: Supplementary Data 6
Description: Sectoral benefit for each country under all combinations of different sets of scenarios.
